# Why physical activity matters for older adults in a time of pandemic

**DOI:** 10.1186/s11556-020-00249-3

**Published:** 2020-09-23

**Authors:** Conor Cunningham, Roger O’ Sullivan

**Affiliations:** 1 Institute of Public Health, City Exchange, 11-13 Gloucester St, Belfast, BT1 4LS Northern Ireland; 2 Ulster University, Newtownabbey, UK

**Keywords:** Physical activity, Older adults, Public health, COVID-19

## Abstract

**Background:**

The COVID-19 pandemic has impacted communities across the world. Government responses, of promoting ‘social distancing’ at a population level, and ‘self-isolation’ of older adults to mitigate its spread have been unprecedented. Despite the importance of these Public Health and Social Measures (PHSM), they present challenges to maintaining a physically active lifestyle, particularly for older adults.

**Context:**

The importance of physical activity (PA) for health is well documented. There is strong evidence that PA in later life reduces the risk of disease, helps to manage existing conditions, and develops and maintains physical and mental function. Staying physically active is particularly important for older adults currently.

**Implications and recommendations:**

Research suggests that PHSM have already reduced levels of PA for older adults during the COVID-19 pandemic. Prior to COVID-19 many older adults were not engaging in enough PA to attain health benefits. Evidence indicates that there will be an increase in the number of older adults not meeting guidelines for PA due to the impacts of COVID-19. This has implications for population health and public health policy. How to support older adults to remain physically active during and after the COVID-19 crisis will require careful consideration. Going forward it is imperative that policy and practice support all older adults to achieve the recommended levels of PA to ensure that they are not disadvantaged in the short- but also in the longer term by the impact of COVID-19.

## Background

There is strong evidence that PA contributes to increased physical function, reduced impairment, independent living, and improved quality of life in both healthy and frail older adults [1]. This stage of life represents an important period to promote PA to improve functions of daily living and slow progression of disease and disability. However, physical inactivity remains a stubborn issue and global progress to increase PA across the life course has been slow [2].

## Context

### Inactivity: a global public health problem

Physical inactivity is recognized as one of the leading risk factors for overweight, obesity, non-communicable diseases, and chronic conditions. It has been identified as the fourth leading risk factor for global mortality (6% of deaths globally) [3]. An estimated 31% of the world’s population do not meet recommended levels of PA [4]. Globally, physically inactive lifestyles have been estimated to cost (INT) $53.8 billion in direct healthcare costs annually [5].

Older adults are at a particular risk of leading inactive lifestyles. For many, aging is defined by rapid declines in levels of PA, loss of mobility and functional independence, and premature morbidity [6]. The prevalence of physical inactivity in older Europeans (> 55 years) has been reported to range from 5% in Sweden to 29% in Portugal [7]. Prior to the COVID-19 pandemic physical inactivity was one of the most important public health challenges facing older adults globally. Increasing PA levels, particularly among those most inactive, was and still is a public health priority. A key public health message for older adults during the COVID-19 pandemic is to be as active as possible, because inactivity has both acute and chronic implications for older adult’s health.

### PA and older adults in a time of pandemic

Government responses to mitigate the spread of COVID-19 have been unprecedented. Internationally, health authorities are advising their populations to implement behavioural strategies which have changed daily life significantly. Older adults have been advised that they are at a higher risk of more serious and potentially fatal illness associated with COVID-19. The global PHSM for older adults include social distancing and self-isolation. An abrupt reduction in both incidental and planned PA due to social distancing and self-isolation is a concern for older adults, who are typically more inactive than younger populations and more prone to frailty and sarcopenia [8].

Self-isolation of older adults has already had negative consequences on levels of PA (e.g. down 53% in those aged 70+ years [9]) and it is likely that there will be significant physical and mental health implications [10, 11]. A recent review of all published literature has also shown that, compared to their active peers, inactive older adults (aged 60+ years) had increased risk of all-cause mortality, fractures, recurrent falls, and functional limitation. They also experienced poorer aging trajectories, a reduction in quality of life and decreased cognitive functioning [12]. Older adults are a very heterogeneous group, and many who are very well and active prior to directions to self-isolate will find it challenging to stay active. The partial or total interruption to habitual PA can lead to deterioration in several metabolic and functional outcomes in older adults. For example, 3 months of detraining has been shown to produce a decline in functional fitness, mental health, quality of life, cardiorespiratory fitness, and lipid, glycemic and haemodynamic profiles in physically active older adults [13, 14]. Therefore, older adults who were regularly active before the pandemic should aim to maintain or increase their levels of PA to achieve recommended levels where possible, while complying with local and national regulations.

Disruption to usual routines, restriction of movement and separation from work and family and friends means that the networks and habits established to maintain activity levels have changed abruptly. Reduced social and physical contact with others has been shown to cause boredom, frustration, and a sense of isolation [10]. In addition, there is a concern that prolonged home stay may increase sedentary behaviours, such as spending excessive amounts of time sitting, reclining, or lying down (watching television, reading, using mobile devices) leading to an increased risk for, and potential deterioration of chronic health conditions [15, 16] and acute and chronic deconditioning. Evidence suggests that the de-conditioning process (loss of muscle strength and decline in ability to perform activities of daily living) can begin within days of becoming inactive [17]. It has been reported that an increase in sedentary behaviours during the pandemic is associated with a decrease in self-reported physical and mental health [18]. The stress and anxiety that people are experiencing during the pandemic may also impair an individual’s immune defence, and in this regard it is critical that older adults aim to maintain recommended levels of physical activity to help boost immune function and mitigate the deleterious effects that inactivity and social isolation may place on the immune system [19].

The pandemic has strained the capacity of healthcare systems internationally, and with the immediate focus on the management of high numbers of critically ill patients, there is concern for the welfare of those with pre-existing comorbidities. Disruption in access to care and preventive interventions for these higher risk individuals can have significant consequences. Consequently, the COVID-19 pandemic may exacerbate pre-existing conditions such as cardiovascular disease, cancer, and diabetes. Maintaining levels of PA is particularly important for older adults with chronic conditions, because having elevated levels of cardiorespiratory fitness and taking part in regular PA at a moderate to vigorous intensity, can reduce chronic low-grade inflammation and improve various immune markers in several disease states including cancer, cardiovascular disease, diabetes, and obesity [20].

PHSM that restrict older adults’ movements do not necessarily mean that PA must be limited, or that all forms of exercise must be eliminated entirely. Many government policies that enforce social distancing and self-isolation allow for daily outdoor exercise, provided safe physical distancing is upheld. In this regard, dissemination of easily understandable information is critical to ensuring that older people have clear messages and resources on how to stay physically and mentally healthy during the pandemic. When PHSM mandate self-isolation in the home for specific groups of older adults (e.g. 70+ years), PA using safe, simple, and easily implemented activities is both possible and necessary to maintain health and fitness levels. Many home-based activities can be vigorous enough to satisfy the criteria for moderate exercise e.g. sweeping floors, mowing the lawn. Aerobic exercise at home can be facilitated by home exercise equipment (if available) and walking around the home/garden. Growing evidence supports the importance of light intensity activity, a message that is particularly important for those who are currently inactive and/or frailer [12].

Numerous exercises for improving muscular strength and balance can be performed in small spaces, with little or no equipment. Body-weight exercises, such as press-ups, can be used to build/maintain strength and household items such as tin cans can be useful as improvised weights. These exercises can be modified to account for current ability. As with any new activity, gradual introduction and progression are important, and those who have previously been inactive should seek advice from a healthcare professional about adopting PA.

Given the increased time that many older adults are spending at home, it is positive that there are many online resources and programs available. The emergence of home-based exercise platforms, such as online instructor-led classes will be particularly useful for older adults during this time. Social support (even remotely) can be important for adoption and maintenance of PA. In addition, health professionals can use existing telemedicine platforms (e.g. via tablets, phones) to support the delivery of appropriate exercise interventions and physical assessments to older adults who are isolating.

Guidance on PA levels during the pandemic needs adjusted to consider the practical context of PHSM and impacts on the environment that PA will now most likely take place in (i.e. at home) and the specifics of mode, duration, intensity and safety to avoid any type of injury.

National policy to support older adults for PA is essential in this context.

Messaging should emphasise that if you are unwell or have COVID-19, use your energy to get better and do not try to be active. If you develop fever, cough, or shortness of breath, stop PA, and contact your health professional.

## Conclusion

At the time of writing, the COVID-19 pandemic is still spreading globally. It is projected that measures to contain the spread of the virus will need to stay in place for some time, especially for older adults. In the wake of the pandemic, it is likely that the proportion of the older adult population inactive and at risk from disease and disorders related to inactivity will have increased. This will have lasting ramifications for population health and related services. In the short, medium, and long-term, decisions about the care and support provided for older adults both during and after the COVID-19 pandemic will require careful consideration.

## Recommendations

### It is imperative that PA should continue to be promoted during this crisis

Governments, public health agencies, health professionals, and community-based organisations and networks must support older adults to be physically active during COVID-19 to decrease the negative physiological and psychological impact of sedentary behaviours. National policy to support older adults for PA during the COVID-19 pandemic is essential.

### Increasing investment in PA and sport programmes

There is a compelling case (for all Governments) to increase investment in PA and sport programmes and improve existing services to ensure they meet the needs of older adults within the context of COVID-19.

### We need to develop our understanding of the key role that health professionals play in promoting PA to older adults

The importance and dedication of healthcare professionals, and the critical role that they play in society has been propelled to the forefront of the national/global psyche. It is recognised that health professionals are key in promoting PA to older adults. This role will be increasingly important in addressing the fall-out from COVID-19 on older adult’s health.

### Robust research and evidence to inform future policy development and service planning for aging populations is more important than ever

Globally, populations are aging. In the time following the peaks of transmission and easing of the PHSM related to COVID-19, high-quality, systematic research will be critical to advancing individual and public health, and informing future policy making and practice in supporting older adults to participate in more active lifestyles.

## Data Availability

Not applicable.
